# Voghera Sweet Pepper: A Potential Ally against Oxidative Stress and Aging

**DOI:** 10.3390/ijms24043782

**Published:** 2023-02-14

**Authors:** Federica Gola, Ludovica Gaiaschi, Elisa Roda, Fabrizio De Luca, Federica Ferulli, Riccardo Vicini, Paola Rossi, Maria Grazia Bottone

**Affiliations:** 1 Department of Biology and Biotechnology, University of Pavia, Via Ferrata 9, 27100 Pavia, Italy; 2 Laboratory of Clinical and Experimental Toxicology, Pavia Poison Centre—National Toxicology Information Centre, Toxicology Unit, Istituti Clinici Scientifici Maugeri, IRCCS Pavia, 27100 Pavia, Italy; 3 Bio Basic Europe S.R.L., Via Taramelli 24, 27100 Pavia, Italy

**Keywords:** *Capsicum annuum* L., aging, oxidative stress, mitochondrial dysfunction, antioxidants

## Abstract

In the present study, the potential functional properties of the extracts from the edible part of *Capsicum annuum* L. var. Peperone di Voghera (VP) were studied. The phytochemical analysis revealed a high amount of ascorbic acid, paralleled by a low carotenoid content. Normal human diploid fibroblasts (NHDF) were chosen as the in vitro model models to investigate the effects of the VP extract on oxidative stress and aging pathways. The extract of Carmagnola pepper (CP), another important Italian variety, was used as the reference vegetable. The cytotoxicity evaluation was performed firstly, using a 3-(4,5-dimethylthiazolyl-2)-2,5-diphenyltetrazolium bromide (MTT) assay, while the VP potential antioxidant and antiaging activity was investigated by immunofluorescence staining focusing on specifically selected proteins. The MTT data revealed the highest cell viability at a concentration of up to 1 mg/mL. The immunocytochemical analyses highlighted an increased expression of transcription factors and enzymes involved in redox homeostasis (Nrf2, SOD2, catalase), improved mitochondrial functionality, and the up-regulation of the longevity gene SIRT1. The present results supported the functional role of the VP pepper ecotype, suggesting a feasible use of its derived products as valuable food supplements.

## 1. Introduction

Aging can be defined as the decline of functional properties at the cell, tissue, and organ level. Individuals are extremely heterogeneous in the onset of the aging process (e.g., rate and extent to which it progresses), indeed both genes and environment influence the functional capacity, the measure of the ability of cells, tissues, and organs to operate optimally [1]. The free radical aging theory is well established among those explaining the aging process [2]. This hypothesis assumes that aging is the result of failure of defensive mechanisms in response to the damage caused by reactive oxygen species (ROSs), particularly at the level of mitochondria [3]. ROSs are generated within the biological system to modulate cellular activities. The increase in ROSs has been associated, despite not being the unique factor, with the onset and progression of aging through oxidative damage and interaction with mitochondria [4]. To minimize the damaging effects of ROSs, different oxidative stress response pathways can be involved. Nrf2, also known as nuclear factor erythroid 2-related factor 2, is a key transcription factor for genes responsive to oxidative stress [5]. Under homeostatic conditions, the Nrf2′s Neh2 domain, which is the main interactor with transcription inhibitor factors such as Keap1, is ubiquitinated and degraded by proteosomes; on the other hand, when the cells are under oxidative stress conditions, the interaction of Keap1 and Nrf2 is disrupted, leading to the upregulation of Nrf2-mediated gene expression [6]. General antioxidant pathways induced by NRF2 comprise enzymes including superoxide dismutase (SOD) and catalase (CAT) [5]. SODs and CAT represent front-line antioxidant defenses protecting against scavenging superoxide radicals and hydrogen peroxide, converting them to less reactive species [7]. Maintaining a low level of ROS within the different cellular compartments is essential for the proper performance of redox-dependent processes, for example, the tricarboxylic acid (TCA) cycle in the mitochondria. The TCA cycle requires, among the others, the activity of the Aconitase 2 (Aco2), a 4Fe-4S iron-sulfur cluster containing enzymes involved in the conversion of citric acid into isocitric acid, and a recent study reported its inhibition under oxidative stress condition [8].

Oxidative damage depends on inherited or acquired defects in enzymes involved in redox reaction; hence antioxidants are assuming a leading role in the control of ROS propagation and formation, subsequently reducing oxidative stress, improving immune functions, and increasing healthy longevity [9]. Indeed, age-related diseases are linked to structural changes in the mitochondria, associated with alterations in the properties of their membrane, from which mitochondrial failure compromises cellular homeostasis and increases vulnerability to oxidative stress [10]. Elderly people are sensitive to oxidative stress due to decreased efficacy of their endogenous antioxidant systems [11,12]. This explains the great efforts devoted by academic and industrial sectors to discover bio-based ingredients able to protect cells from ROS-induced damage.

For decades, the consumption of a fruit- and vegetable-rich diet has been considered salubrious, increasing longevity and decreasing morbidities. Recent interest in the use of complementary and alternative medicine, using natural products, has increased considerably to improve the health and well-being of humans [13,14]. In particular, the antioxidant properties of vitamin E, polyphenols, and flavonoids, as beneficial components from fruit and vegetables, have often been claimed to be responsible for the protective effects against cardiovascular disorders, cancer, and neurodegenerative diseases, being able to better quality of life and longer life expectancy [15,16]. To date, many studies have demonstrated that polyphenols and flavonoids have the antioxidant effect of scavenging ROS, able to enhance the autophagy process [13,14,17]. These valuable effects could be crucially employed to support healthy aging. This bulk of the literature data provide the basis for the current increasing interest in the use of natural antioxidants as functional food ingredients and/or as food supplements [18]. Nonetheless, the correct identification of the active ingredients and their amounts, together with assessment of daily intake, still remains a critical bottleneck for translating observational epidemiological data to the actual development/use of safe functional foods/supplements in everyday diet.

Traditional plant/vegetable varieties are those that have been distinguished by farmers through a historical breeding process [19]. They thus represent an important genetic patrimony as a source of agricultural biodiversity, which is a key element to guarantee the quality of food [20].

Peppers are vegetables belonging to *Capsicum annuum*, the most economically important genus of the Solanaceae family from South America [21]. In Italy, the multiple climatic conditions favored the collection of numerous local cultivars: a typical example is the “Voghera pepper”. It represents a small local reality, which is cultivated in the province of Alessandria and Pavia by a limited number of farmers and producers [22]. The Voghera pepper disappeared from our diet in the early ‘50 s due to a disease that compromised its commercial production. In 2006, it was reintroduced, and it has begun to be cultivated again [23].

The consumption of pepper is constantly growing. The high nutritional value of this food, rich in vitamin C, vitamin E, and carotenoids, is widely recognized by the scientific literature [24,25,26].

In particular, ascorbic acid and carotenoids were reported for their role in protection against oxidative damage and cell aging [27,28].

Thus, the aim of the current investigation was to assess the biological activity, evaluated in terms of antioxidant and antiaging effects, of Voghera pepper extract, comparing it with the Carmagnola pepper extract, another important Italian variety, used as the reference vegetable. The study was conducted using young and old primary normal human dermal fibroblasts (NHDFs), firstly evaluating the cell viability after exposure to the two different pepper extracts and then assessing, by immunofluorescence, several proteins, i.e., Nrf2, SOD2, catalase, COX4, Aco2, COX2, and SIRT1, as representative markers of oxidative stress and age-related pathways.

## 2. Results

### 2.1. Chemical Characterization

The nutraceutical profiles of both Voghera pepper (VP) and Carmagnola pepper (CP) were determined by the measurement of carotenoids and ascorbic acid (Table 1).

In both extracts, carotenoids were detected, including carotene derivatives, α-carotene, β-carotene, lutein, and zeaxanthin.

The number of total carotenoids was much higher in the extract of CP (+103.07%); nonetheless, it has to be underlined that in the VP a higher quantity of the xanthophyll lutein was determined (+45.22%).

On the other hand, the VP extract contains a higher amount of ascorbic acid (+91.33%). Moreover, a minimum amount of dehydroascorbic acid was detected in both extracts.

### 2.2. Validation of the Replicative Senescent Model

**Figure 1:**
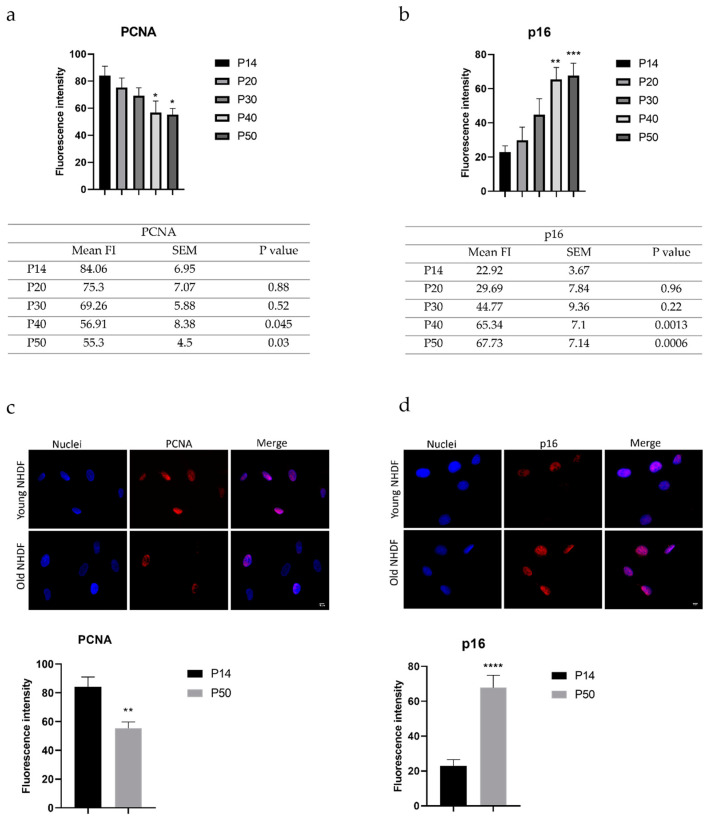
Histograms and tables showing data concerning the two chosen markers of proliferation or aging: (**a**) PCNA and (**b**) p16, evaluated at selected culture passages, i.e., passages 15, 20, 30, 40, and 50 (data expressed as mean of fluorescence intensity ± standard error of the mean); * *p* < 0.05, ** *p* < 0.01, *** *p* < 0.001, ****p < 0.0001 compared to P14 as determined by one-way Anova. (**c**) Immunofluorescent labeling for PCNA protein (in red); DNA was stained with Hoechst 33258 (blue fluorescence). Magnification 60×; scale bar: 10 μm. The histograms show the fluorescence intensity value of the immunolabeling in young (passage 14) and old (passage 50) NHDF. ** Student’s *t*-test: *p* < 0.01; (**d**) Immunofluorescent labeling for p16 protein (red fluorescence); DNA was stained with Hoechst 33258 (blue fluorescence). Magnification 60×; scale bar: 10 μm. The histograms show the fluorescence intensity value of the immunolabeling in young (passage 14) and old (passage 50) NHDF. **** Student’s *t*-test: *p* < 0.001.

To establish after how many passages in culture the NHDF can be defined as aged fibroblasts, NHDF were studied at precise culture time points (i.e., passages 15, 20, 30, 40, and 50) at the beginning of our experimental design, with the goal to establish the ideal number of passages to be chosen. Parallelly, two markers, i.e., Proliferating Cell Nuclear Antigen (PCNA) and tumor suppressor protein p16INK4a (CDKN2A, p16), namely p16, representative of proliferation and senescence, respectively, were analyzed at the above-reported culture passages (by immunofluorescence staining) to characterize the features of cells under investigations, and, thus, properly identify aged fibroblasts. Figure 1a,b illustrates data concerning PCNA and p16 at the selected culture passages.

As shown in Figure 1 (micrographs and c), PCNA was identified in the nucleus. In young NHDF, an intense signal was evidently observed, indicating the presence of active proliferating cells. In the old NHDF sample, a significant decrease in fluorescence intensity was determined (*p* = 0.0024; 84.06 ± 6.95 vs. 55.3 ± 4.5, for young and old NHDF, respectively).

Figure 1 (micrographs and d) illustrates the immunofluorescence analysis of p16 expression. p16 was localized mainly in the nucleus, and its fluorescence intensity increased significantly in NHDF at passage 50 (*p* < 0.0001; 22.92 ± 3.67 vs. 67.73 ± 7.14 for young and old NHDF, respectively). Based on the above-reported data, passage 50 was identified as the proper culture timepoint to define NHDF as aged fibroblasts.

### 2.3. MTT Assay

**Figure 2:**
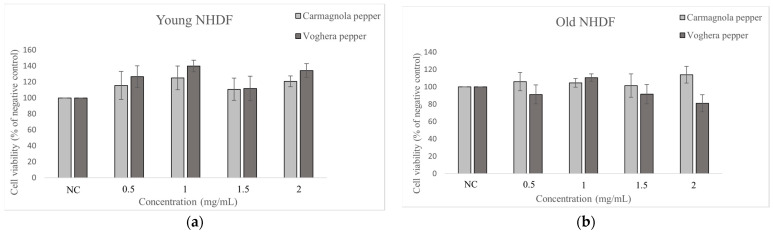
Cell viability after 24 h-exposure to different concentrations of two different pepper extracts, i.e., VP or CP: (**a**) Young NHDF; (**b**) Old NHDF. Values are mean ± SEM; Anova one-way test: not statistically significant.

The cytotoxicity of both pepper extracts was tested by MTT assay. Figure 2 shows the viability, expressed as percentage (%) of the negative control, of young (a) and old (b) NHDF exposed to different concentrations of VP or CP extracts (tested dose range: 0.5; 1; 1.5; 2 mg/mL) for 24 h. The viability of young fibroblasts was generally increased with the addition of either VP or CP extracts; in particular, a viability of 125% and 139% was measured in CP and VP-exposed cells, respectively, at the concentration of 1 mg/mL. Despite that the increase in viable cells was not statistically significant compared to the negative control (CP: *p* = 0.66; VP: *p* = 0.11), the viability data suggested the concentration to be used for subsequent studies. Although we observed a decreased viability in old fibroblasts compared to the young ones, consistent with the results previously shown, at the concentration of 1 mg/mL, the viability was >100% for both extracts (104.6% vs. 110.7%), albeit without statistical significance compared to the negative control (CP: *p* = 0.82; VP: *p* = 0.22).

The quantitative analyses also measured a non-statistically significant difference among groups exposed to different doses of pepper extracts, i.e., VP or CP (comparing treated NHDF with the negative control), revealing *p* values ≥ 0.05. The lack of statistical significance was determined both in young and old NHDF.

### 2.4. Antioxidant Activity

**Figure 3:**
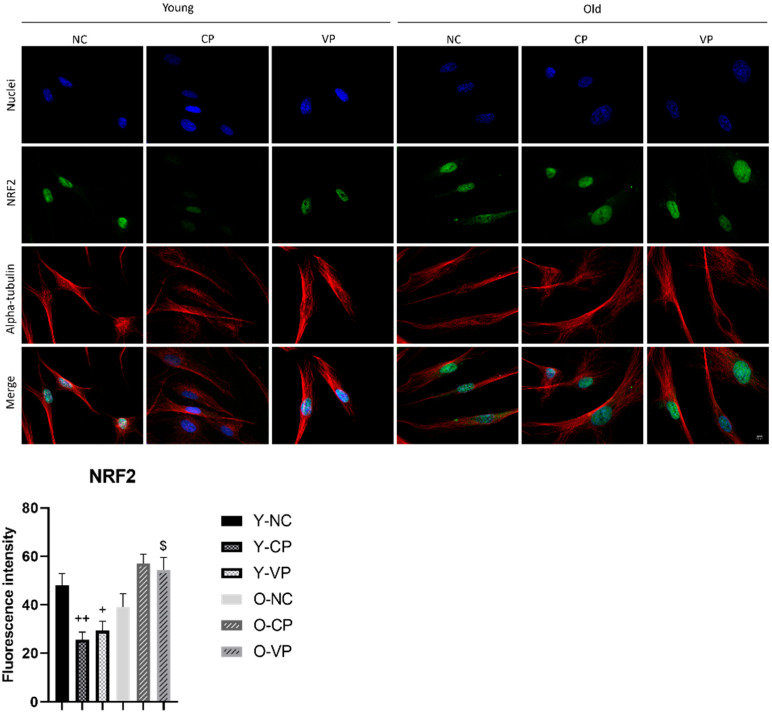
Double immunolabelling for Nrf2 protein (green fluorescence) and alpha-tubulin (red fluorescence); DNA was stained with Hoechst 33258 (blue fluorescence). Magnification: 60×; scale bar: 10 μm. Histograms representing the analysis of the fluorescence intensity of Nrf2; Anova one-way test: *p* < 0.0001; ++ *p* < 0.01, statistical significance between untreated young NHDF (Y-NC) and young NHDF treated with CP extract (Y-CP); + *p* < 0.05, statistical significance between young NHDF treated with VP and negative control; $ *p* < 0.05, statistical significance between old NHDF treated with VP extract and old NHDF untreated.

The effect of peppers on Nfr2 in young and aged fibroblasts was primarily evaluated. As depicted in Figure 3, the results showed an increase of Nfr2 in old NHDF samples treated with the pepper extracts compared to old and young NHDF controls (samples without addition of any extract). Young fibroblasts treated either with VP or CP showed a decrease in Nfr2 expression compared to control young fibroblasts. This data confirmed that in conditions of cellular homeostasis, the activity of Nfr2 is inhibited. Our analyses revealed that Nrf2 had a decreasing trend, even though not statistically significant (*p* > 0.999), in the old NHDF negative control (O-NC) compared to the young NHDF negative control (Y-NC) (48.0182 ± 4.9 vs. 39.15 ± 5.4). A significant decrease in Nrf2 expression was determined by comparing treated young samples with the young controls (Y-NC vs. CP: *p* = 0.0059, 48.02 ± 4.97 vs. 25.55 ± 3.2; Y-NC vs. VP: *p* = 0.03, 48.02 ± 4.97 vs. 29.28 ± 3.96). In addition, we observed an increase of Nrf2, albeit without a significant difference, in old NHDF treated with VP (O-VP) compared to the old negative control (O-NC) (*p* = 0.14; 39.15 ± 5.4 vs. 54.34 ± 5.26).

**Figure 4:**
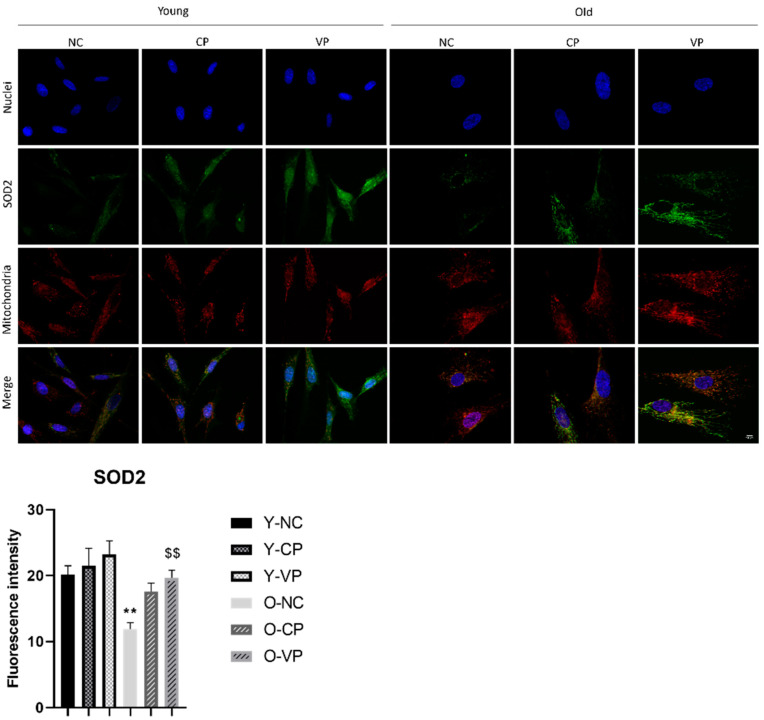
Double immunolabeling for SOD2 protein (green fluorescence) and mitochondria (red fluorescence); DNA was stained with Hoechst 33258 (blue fluorescence). Magnification: 60×; scale bar: 10 μm. Histograms representing the analysis of the fluorescence intensity of SOD2; Anova one-way test: *p* = 0.02; ** *p* < 0.01, statistical significance between untreated young NHDF (Y-NC) and old untreated NHDF (O-NC); $$ *p* < 0.01, statistical significance between old NHDF treated with VP and old NHDF untreated.

Then, we assessed SOD2 expression (Figure 4), revealing a significant decrease in the old NHDF negative control (O-NC) compared to the young one (Y-NC) (*p* = 20.13 ± 1.38 vs. 11.88 ± 0.99). Although any significant difference was measured, an increase in SOD2 expression was evident when comparing young samples treated with VP with the young negative control (20.13 ± 1.38 vs. 23.24 ± 2.05). Moreover, a significant increase of this enzyme was determined in old NHDF treated with the VP extract (O-PV) compared to the old negative control (*p* = 0.01; 11.88 ± 0.99 vs. 19.65 ± 1.16).

**Figure 5:**
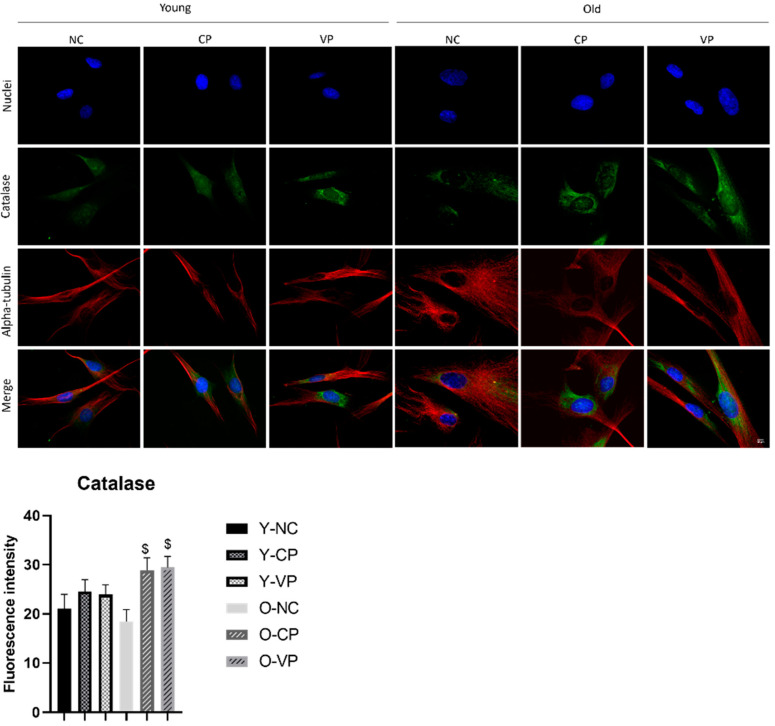
Double immunolabeling for protein catalase (green fluorescence) and α-tubulin (red fluorescence); DNA was stained with Hoechst 33258 (blue fluorescence). 60× magnification; scale bar: 10 μm. Histograms showing the analysis of catalase fluorescence intensity; Anova one-way test: *p* = 0.01; $: *p* < 0.05, statistical significance between old NHDF treated and old NHDF untreated.

Next, we focused on the other antioxidant enzyme, namely catalase. Figure 5 depicted the observed decrease in catalase expression in the old negative control compared to the young one, albeit any significant difference was determined. Moreover, a general increase in catalase was assessed by comparing treated young samples with the young negative control, even though any significant difference was measured. Lastly, we revealed a significant increase of catalase in old NHDF exposed to the VP extract (O-VP) compared to the old untreated control (O-NC) (*p* = 0.02; 18.37 ± 2.48 vs. 29.44 ± 2.27).

To further evaluate the impact of VP extract on the cellular antioxidant response, we investigated the expression pattern of Cyclooxygenase 4 (COX4) and Aconitase 2 (Aco2).

**Figure 6:**
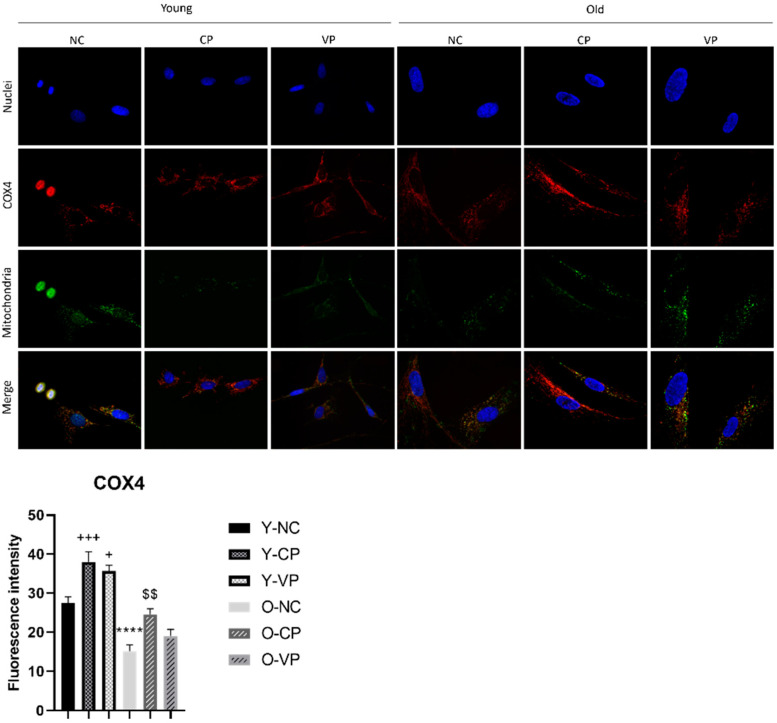
Double immunolabeling for protein COX4 (red fluorescence) and mitochondria (green fluorescence); DNA was stained with Hoechst 33258 (blue fluorescence). 60× magnification; scale bar: 10 μm. Histograms showing the analysis of COX4 fluorescence intensity; Anova one-way test: *p* = 0.01; **** *p* < 0.0001, statistical significance between untreated young NHDF (Y-NC) and old untreated NHDF (O-NC); +++ *p* < 0.001, statistical significance between untreated young NHDF (Y-NC) and young NHDF treated with CP extract (Y-CP); + *p* < 0.05, statistical significance between young NHDF treated with VP and negative control; $$ *p* < 0.01, statistical significance between old NHDF treated with CP and old NHDF untreated.

Figure 6 illustrates the localization of COX4 which resulted evidently restricted in the mitochondria region, at all experimental conditions, both in young and old fibroblasts; notably, an altered mitochondrial morphology was revealed in old fibroblasts, which appeared enlarged and more elongated than in young fibroblasts. COX4 expression levels decreased significantly in the old NHDF control (O-NC) compared to the young NHDF control (Y-NC) (*p* < 0.0001; 27.49 ± 1.58 vs. 15.15 ± 1.66). Furthermore, COX4 increased significantly in young NHDF exposed to CP (Y-CP) and VP (Y-VP) extracts (*p* = 0.0006, 27.49 ± 1.58 vs. 38.03 ± 2.57; *p* = 0.01, 27.49 ± 1.58 vs. 35.73 ± 2.57). Although not statistically significant, an increase in COX4 expression levels was observed in the O-VP sample (19.06 ± 1.66 vs. 15.15 ± 1.66).

**Figure 7:**
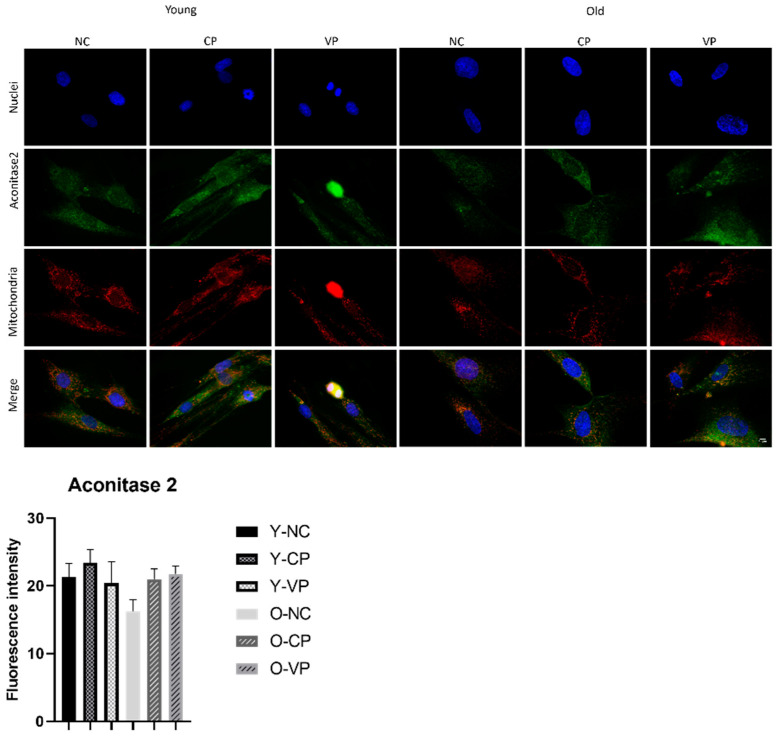
Double immunolabeling for Aco2 (green fluorescence) and mitochondria (red fluorescence); DNA was stained with Hoechst 33258 (blue fluorescence). 60× magnification; scale bar: 10 μm. Histograms represent the analysis of Aco2 fluorescence intensity. Anova one-way test: not statistically significant.

Next, our investigation focused on Aco2. Figure 7 illustrates the Aco2 expression pattern. Aco2 appeared to be diffusely localized in the cytoplasm and partially in the mitochondria at all experimental conditions. A decrease of Aco2 expression levels was detected comparing young NHDF control (Y-NC) with old NHDF control (O-NC) (21.30 ± 2.01 vs. 16.27 ± 1.73), although the quantitative analysis revealed difference values not statistically significant. Furthermore, an increase in Aco2 expression levels was observed in old NHDF exposed to VP (O-VP) or CP extract (O-CP) compared to the old NHDF control (O-NC) (respectively: 16.27 ± 1.73 vs. 21.76 ± 1.18; 16.27 ± 1.73 vs. 20.99 ± 1.51); even in this case, the quantitative determinations revealed a lack of statistical significance.

**Figure 8:**
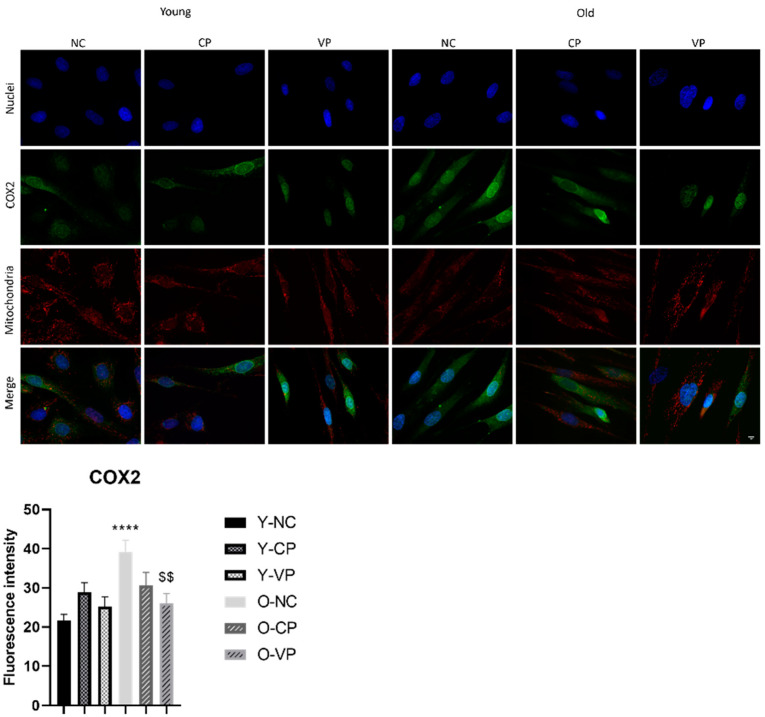
Double immunolabeling for COX2 (green fluorescence) and α-tubulin (red fluorescence); DNA was stained with Hoechst 33258 (blue fluorescence). 60× magnification; scale bar: 10 μm. Histograms showing the analysis of COX2 fluorescence intensity; Anova one-way test: *p* = 0.0003; **** *p* < 0.0001, statistical significance between old NHDF untreated and young NHDF untreated; $$ *p* < 0.01, statistical significance between old NHDF treated with VP and old NHDF untreated.

Further, COX2 was investigated as an indicator of oxidative stress/inflammation. This protein was localized both in the cytoplasm and in the nuclear membrane (Figure 8). In line with previous studies, we revealed that COX2 expression increased significantly in the old NHDF control (O-NC) compared to the young NHDF control (Y-NC) (21.60 ± 1.64 vs. 39.25 ± 2.94). Additionally, an increase of COX2 expression levels was observed comparing treated young samples and the NHDF (Y-NC) control, although the quantitative analysis did not give statistically significant values. Finally, a significant COX2 decrease was measured in old NHDF exposed to VP extract (O-VP) (39.25 ± 2.94 vs. 26.07 ± 2.44); similarly, a COX2 decrease was revealed in the old NHDF sample treated with the CP extract (O-CP) compared to the old control (*p* = 0.0048; 39.25 ± 2.94 vs. 30.58 ± 3.35)

### 2.5. Evaluation of the Antiaging Properties

**Figure 9:**
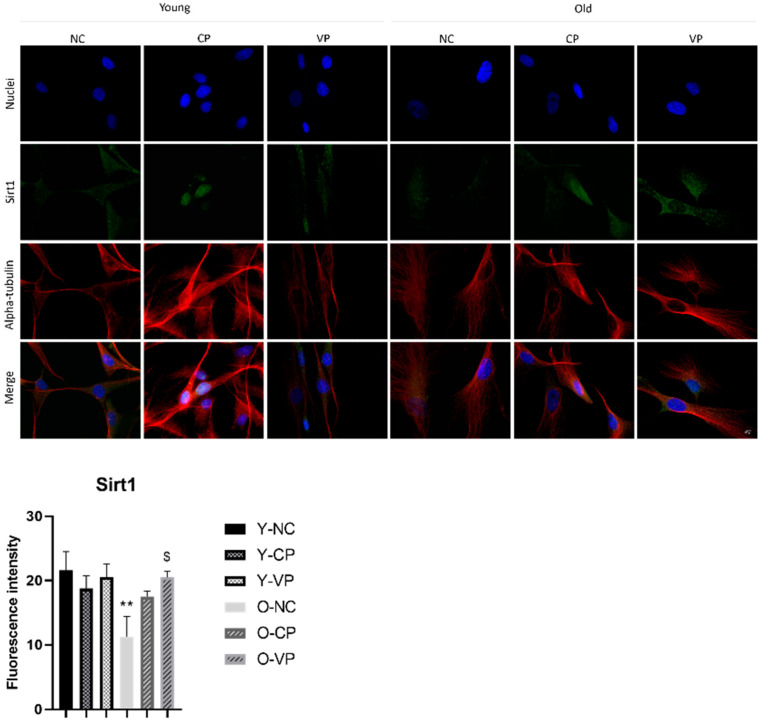
Double immunolabeling for SIRT1 (green fluorescence) and α-tubulin (red fluorescence); DNA was stained with Hoechst 33258 (blue fluorescence). 60× magnification; scale bar: 10 μm. Histograms representing the analysis of SIRT1 fluorescence intensity; Anova one-way test: *p* = 0.02; ** *p* < 0.01, statistical significance between old NHDF untreated and young NHDF untreated; $ *p* < 0.05, statistical significance between old NHDF treated with VP and old NHDF untreated.

To investigate the aging pathway, we assessed the expression levels of SIRT1 since it possesses a pivotal role in the regulation of aging mechanisms and lifespan. Figure 9 depicted the expression pattern of SIRT1, which appeared mainly located in the nucleus. However, especially in old treated fibroblasts (either exposed to VP or CP extract), cytoplasmic labeling was also detectable. In the control condition, young cells exhibit a strong immunopositivity for SIRT1, while in the old control SIRT1 expression levels significantly decreased (*p* = 0.009; 21.57 ± 2.92 vs. 11.23 ± 3.16). The exposure to pepper extracts in old NHDF seemed to impact SIRT1 expression since its levels increased compared to the old negative control. In particular, the measurement of SIRT1 fluorescence intensity demonstrated a significant enhancement in old NHDF exposed to the VP extract (O-VP) compared to the old NHDF control (O-NC) (*p* = 0.03; 11.23 ± 3.16 vs. 20.48 ± 0.94).

## 3. Discussion

Aging is defined as the natural result of entropy in animal cells, tissues and organs. In particular, aging, characterized by an advancing loss of physiological integrity and leading to impaired function/enhanced vulnerability to death, is crucially related to oxidative stress and chronic inflammation [1,29]. Therefore, great effort is currently devoted to the search for new therapies able to slow down the processes of cognitive and motor decline linked to physiological aging. Antioxidants can modulate the inflammatory state, hence playing an essential role in protecting cells against oxidative stress-related damage by eliminating excessive free radicals [30]. Over the past decade, with the aim of improving healthy aging, several supplements containing food-derived antioxidants have been introduced in the field of preventive medicine as adjuvant anti-senescence compounds. Actually, the effect of this type of oral supplementation has been shown to significantly increase the antioxidant defense system [30].

Aging is characterized by a number of mechanisms underlying typical processes, such as cell death, genomic instability, telomere shortening, protein synthesis deregulation and degradation, microbiota disorders, mitochondrial dysfunction, oxidative stress, and *inflammaging* [31]. Our current findings demonstrated the antioxidant and anti-aging properties of the VP. In particular, the characterization of the VP extract revealed the presence of an ascorbic acid concentration about two-time fold higher compared to that measured in the reference pepper, namely the Carmagnola pepper (CP). Several literature data demonstrated that ascorbic acid is able to reduce mitochondrial and DNA damage by lowering ROS [32]. Moreover, in the VP a higher concentration of lutein was determined; lutein is a xanthophyll known for its antioxidant and protective properties on vision, known to be effective also as anti-aging compound, since it seems to slow down the oxidative processes responsible for structural and functional aging in numerous organs and tissues [33].

The chosen in vitro model, i.e., young and old fibroblasts (NHDF), employed to test the pepper extracts was characterized to mimic physiological aging, as supported by the data obtained through the evaluation of two peculiar parameters, (i) PCNA, used to assess the proliferation rate, and (ii) p16, employed to demonstrate permanent cell cycle arrest with the appearance of the senescent phenotype [34,35,36].

Based on the MTT assay results, the young and old fibroblasts were exposed for 24 h to either VP or CP extract at the dose of 1 mg/mL; at this concentration, striking viability was assessed in treated fibroblasts, even exceeding 100%.

MTT assay is one of the often-used cell viability/cytotoxicity assays, and viabilities above 100 % are not unusual. We hypothesized that this data can be justified based on two main explanations: (i) the random experimental fluctuation (usually within +/− 10%), and (ii) the stimulation induced by the treatment, namely hormesis. MTT is prone to compounds interfering with energy metabolism, which can increase MTT data to up to % baseline activity; on the other hand, viability data exceeding 100% are measured when cells under investigation are proliferating or metabolizing MTT more than the control. Notably, in our current study, viability above 100% was measured in young fibroblasts, after exposure to either VP or CP extracts at the dose of 1 mg/mL (125% and 139% measured in CP and VP-exposed cells, respectively). Notably, even though a decrease was assessed compared to young NHDF, viability higher than 100% was determined in old NHDF after treatment with pepper extracts. Hence, in line with the previous literature regarding a wide range of plant extracts [16,37], these findings support the notion that a 1 mg/mL concentration of VP extract was even able to trigger fibroblast cell proliferation [38,39].

The oxidative stress theory is based on the hypothesis that the functional losses associated with aging are due to the accumulation of damage induced by reactive oxygen and nitrogen species (RONS) [40]. The nuclear factor erythroid-derived 2-like 2 (Nrf2) is the key regulator of the protective mechanism against oxidative stress; it controls the basal and induced expression of many antioxidant response element-dependent genes to regulate the physiological and pathophysiological outcomes of oxidant exposure [35]. The Nrf2 cellular expression declines during aging; thus, Nrf2 is considered a promoter of aging, and its loss of function impairs proteasome assembly, resulting in an accumulation of misfolded proteins. Nrf2 has also a crucial role in autophagy; indeed, it regulates several ATG proteins that control autophagosome formation, inducing a related decrease in the autophagic process [41,42].

Furthermore, the age-related lessening of Nfr2 leads to both genomic instability and inhibition of DNA damage-repairing processes, with a consequent increase in the production of ROS by the aging cell [35].

Evidence from the literature demonstrated that Nfr2 maintains redox homeostasis and exerts anti-inflammatory and antitumor activity [43] and, in non-stress conditions, it maintains relatively low cellular values, in accordance with our results in young control fibroblasts. Diversely, we revealed it decreased in old control fibroblasts, but notably, after exposure to both tested pepper extracts (either VP or CP) Nrf2 expression levels significantly enhanced. Considering the role of Nfr2 in mediating the antioxidant response, we therefore studied the expression of other proteins which are considered key antioxidant markers: SOD2, catalase, aconitase, COX4, and COX2 in young and old fibroblasts in culture with and without the addition of VP extract. Our results demonstrated that exposure to either VP or CP extracts increased the expression of Nfr2 and all other investigated antioxidant proteins in old fibroblasts. In particular, SOD2 and catalase resulted significantly augmented.

To further investigate the oxidative stress pathway, the enzymes COX4 and Aco2 were then assessed.

COX4 is the terminal electron acceptor of the mitochondrial respiratory chain which catalyzes the transfer of electrons from cytochrome c to molecular oxygen, contributing to the electrochemical gradient used by ATP synthase to form ATP [44]. COX4 showed an upward trend demonstrating that the exposure to VP extract improves the cellular respiration process and parallelly decreases oxidative stress.

Aco2 is an enzyme involved in the conversion of citric acid into isocitric acid in the tricarboxylic acid cycle. Ciccarone and colleagues reported that the reduction of Aco2 seemed to be linked to mitochondrial dysfunction, with the release of H_2_O_2_ ions and increased oxidative stress [45]. In our study, we observed an increase in Aco2 expression in old NHDF treated with the VP, indicating that treatment with the extract may restore age-related decline of mitochondrial function, hence helping to slow down the aging process.

Finally, COX2 is an enzyme involved in stress responses, and its activity can produce oxidative damage, which suggests that it may contribute to the senescence process. It has been shown that COX2 expression increases in the senescence state of normal human dermal fibroblast cells compared to young NHDF cells [46]. Indeed, we observed that COX2 expression enhanced significantly in the old non-treated fibroblasts compared to the young ones; a significant reduction of COX2 expression levels was observed in old NHDF in both experimental groups (fibroblasts treated either with VP or CP extracts). These results highlighted that treatment with the two pepper extracts (either VP or CP) could reduce COX2 expression levels related to aging, thus decreasing oxidative damage.

As concerns to the aging pathway, we investigated the expression levels of SIRT1 since sirtuins are known to mediate cell aging by delaying senescence or by expanding cell lifespan through the regulation of various cellular functions [47]. In particular, SIRT1 is an NAD+-dependent histone/protein deacetylase responsible for regulating physiological and metabolic responses to stress signals. Therefore, SIRT1 plays a critical role in cell survival and protects the cell against aging [47,48] since it directly defends against oxidative stress and modulates inflammatory responses [48]. Our current findings demonstrated a significant upregulation in the expression of SIRT1 in old NHDF cells treated with VP extract compared to the negative control. This data is in line with previous literature findings [49,50].

Taken together, our results indicated that VP is able to lead the activation of Nrf2 which functions as a central regulator of oxidative stress, by modulating other molecular factors involved in oxidative balance mechanisms and age-related processes. It has to be considered that, under normal physiological conditions, Nrf2 is bound to its inhibitory protein actin Keap1 in the cytosol, while, in response to oxidative stress, Nrf2 is released from Keap1 and translocates to the nucleus [51]. Furthermore, active Nrf2 levels are regulated by autophagy and ubiquitin-binding protein p62 [52]. Therefore, we plan to investigate the role of VP both on Keap1 and the autophagic process, to understand its potential anti-aging effect.

## 4. Materials and Methods

### 4.1. Extracts Preparation and Characterization

Extraction procedure was performed as follows: 15 g of pepper were left to macerate in 100 g mixture water/1,2-propanediol (45% water−55% 1,2-propanediol) × 2 h at 40 °C, then the sample was subjected to homogenization for 15 min and placed to macerate at 40 °C for another 2 h. The sample was left to rest for 24 h in order to decant the solid part and the overlying part was taken. For the extraction of carotenoids samples were diluted 1:1 in acetone, sonicated in ultrasonic bath, centrifuged, and placed in vial for chromatographic analysis. The samples were analyzed using the HPLC-UV-Vis system at wavelengths of 425 and 450 nm. A YMC Carotenoid 5 μm 4.6 × 250 mm column with a flow of 1.3 mL/min was used as the stationary phase. Methanol and MTBE/methanol were used as moving phases (90:10). Analytical standards of β-carotene, astaxanthin and lutein and previous identifications made in LC-MS were used as a reference. Total carotenoids have been expressed generically as beta carotene. For the extraction of ascorbic acid and DHA the samples were diluted 1:1 in 1% formic water for dehydroascorbic acid analysis and 1:10 in 1% formic acid analysis, sonicated in ultrasonic bath, centrifuged, and placed in vial for chromatographic analysis. The samples were analyzed using the HPLC-UV-Vis system at wavelengths of 254 and 295 nm. An Eclipse XDB C8 5 μm 4.6 × 150 mm column with a flow of 1.1 mL/min was used as the stationary phase. As mobile phases, we used water, 1% formic acid, and methanol in isocratic phase 98: 2 for 10 min. Analytical standards of ascorbic acid and dehydroascorbic acid were used as a reference.

### 4.2. Cell Cultures and Treatments

Human neonatal dermal fibroblasts (NHDF) gently donated by Bio Basic S.r.l were cultured in Dulbecco’s Modified Eagle’s Medium (DMEM) with 10% fetal bovine serum and penicillin/streptomycin (100 IU/50 μg/mL) and maintained in medium in a humidified atmosphere containing 5% CO_2_ in air at 37 °C. Young NHDFs from passages 14 to 19 were used for the experiments. Replicative senescent NHDFs were induced by long-term passaging of the cells in cell culture. Cells acquired the senescence phenotype at passage 50. Forty-eight hours before experiments, NHDF were seeded on glass coverslips (200,000 cells) for fluorescence microscopy; young fibroblasts and old fibroblasts (passages 50 to 54) were treated for 24 h with Voghera pepper (VP) extract and Carmagnola pepper (CP) used as the control reference extract, at a concentration of 1 mg/mL.

### 4.3. Cell Viability

Firstly, cytotoxicity of VP and CP was tested using the MTT assay, as valuable indicator of cell viability and proliferation. This colorimetric assay is based on the reduction of a yellow tetrazolium salt (3-(4,5-dimethylthiazol-2-yl)-2,5-diphenyltetrazolium bromide or MTT) to purple formazan crystals by metabolically active cells [53]. Young and senescent NHDFs were seeded at a density of 5 × 10^3^ cells/well on 96-well plates. Cells were incubated with different concentrations (0.5–1–1.5–2 mg/mL) of test materials for 24 h in a humidified incubator at 37 °C. MTT solution (5 mg/mL) was subsequently added to each well and incubated at 37 °C for additional 3 h. Finally, the supernatant was removed and the formazan crystals were dissolved in 100 μL of DMSO, and the absorbance was measured at 490 nm by microplate reader. The optical density of the formazan formed in the control group cells was taken as 100%. Cell viability % was calculated as follows: (Absorbance value of treated cells − Absorbance value of blank)/(Absorbance value of untreated cells − Absorbance value of blank)} × 100 [54].

### 4.4. Immunofluorescence Reactions

Control and treated cells were fixed with 4% formalin for 20 min and post-fixed with 70% ethanol at −20 °C for at least 24 h. Samples were rehydrated for 10 min in PBS and then unspecific sites were blocked by using a blocking solution of PBS supplemented with 4% BSA and 0.2% tween for 15 min RT. Next, cells were immunolabeled with primary antibodies diluted in PBS-Tween 0.2% for 1 h, at RT in a dark moist chamber. After 3 washes in PBS of 5 min each, coverslips were incubated with secondary antibodies in in PBS-Tween 0.2% for 45 min. At the end of incubation, sections were counterstained for DNA with 0.1 µg/mL Hoechst 33258, washed with PBS, and mounted in a drop of Mowiol, for fluorescence microscopy analysis [55,56]. Primary antibodies used for immunofluorescence reactions are reported in Table 2. An Olympus BX51 microscope equipped with a 100-W mercury lamp was used under the following conditions: 330–385 nm excitation filter (excf), 400 nm dichroic mirror (dm) and 420 nm barrier filter (bf) for Hoechst 33258; 450–480 nm excf, 500 nm dm and 515 nm bf for the fluorescence of Alexa 488; 540 nm excf, 580 nm dm and 620 nm bf for Alexa 594. Images were recorded with an Olympus MagniFire camera system and processed with the Olympus Cell F software.

### 4.5. Immunofluorescence Quantification

After immunocytochemical reactions, images acquisition was performed by Cell F software. To make the fluorescence intensity comparable, during image acquisition the exposure time to detect every single fluorescence was selected based on the control sample and then maintained constant for the respective experimental conditions, thus avoiding the insertion of any variables in the analysis. The fluorescence intensity of the proteins of interest was analyzed with the ImageJ software. The channels of each fluorescence have been split to obtain the single images in a greyscale where the minimum value is 0 (black) and the maximum value is 255 (white) [55,56].

### 4.6. Statistical Analyzes

For immunofluorescence quantification 11 quadrants were evaluated for a random analysis and the values obtained were expressed as the mean ± SEM [56]. Data were analyzed using Student *t* test to determine differences between two variables and one-way ANOVA with Bonferroni post hoc comparison test (GraphPad Prism 8.0.1) to compare differences among more than two variables; *p* values less than 0.05 were considered statistically significant.

## 5. Conclusions

Taken as a whole, our current data revealed the absence of cytotoxicity of the VP extract on “young” or “old” fibroblasts nonetheless assessing beneficial effects able to counteract oxidative stress and aging processes. In particular, any alterations of the cytoplasmic organelles or induction of cell death pathways were evidenced in treated young and senescent NHDFs, confirming the efficacy of the VP extract even though the mechanism of action needs to be further elucidated. Ongoing studies aim to evaluate the effects of the VP on genetic, epigenetic, and metabolic alterations typical of aging pathways. Based on this preliminary classification of its antioxidant properties, VP seems to be an excellent product that is usable in the fight against oxidative stress damages, aging, and related pathologies. These findings suggest that VP could be employed as an effective active ingredient in future formulations for food supplements, providing a good start to healthy aging!

## Figures and Tables

**Table 1:** Total carotenoids and ascorbic acid contents in the tested extracts.

Bioactive Compounds (μg/g)	Voghera Pepper (VP)	Carmagnola Pepper (CP)
Carotene derivatives	n.d.	21.8 ± 0.7
α-carotene	11.3 ± 0.2	13.9 ± 0.2
β-carotene	9.6 ± 0.1	21.4 ± 0.7
Trans-β-carotene	n.d.	14.4 ± 0.2
Lutein	16.7 ± 0.2	11.5 ± 0.02
Zeaxanthin	9.6 ± 0.6	15.4 ± 0.1
Zeaxanthin derivatives	n.d.	14.1 ± 0.2
Total carotenoids	55.4	112.5
Ascorbic Acid	724.2 ± 0.37	378.5 ± 0.25
Dehydroascorbic acid (DHAA)	17.5 ± 0.50	13.4 ± 0.44

**Table 2:** Primary antibodies used for immunofluorescence reactions.

Antigen	Primary Antibody	Dilution in PBS
***PCNA***	Mouse monoclonal anti-PCNA (Calbiochem, San Diego, CA, USA)	1:200
***P16INK4a*** ***/*** ***CDKN2A***	Mouse monoclonal anti- p16INK4a/CDKN2A (GeneTex, Irvine, CA, USA)	1:250
***Sirt1***	Rabbit polyclonal anti-Sirt1 (Abcam, Cambridge, UK)	1:100
***SOD2***	Rabbit monoclonal anti-SOD2 (Cell Signaling Technology, Danvers, MA, USA)	1:200
***Catalase***	Rabbit monoclonal anti-Catalase (GeneTex, Irvine, CA, USA)	1:250
***NRF2***	Rabbit polyclonal anti-NRF2 (Abcam, Cambridge, UK)	1:200
***COX4***	Mouse monoclonal [20E8C12] anti-COX4 (Abcam, Cambridge, UK)	1:200
***ACO2***	Rabbit polyclonal anti-Aconitase2 (Abcam, Cambridge, UK)	1:200
***Mitochondria***	Human autoimmune serum recognizing the 70 kDa E2 subunit of pyruvate dehydrogenase complex	1:300
***α-tubulin***	Mouse monoclonal anti-Alpha-tubulin (Cell Signaling Technology, Danvers, MA, USA)	1:1000

## Data Availability

Not applicable.
